# The association between sleep and early pubertal development in Chinese children: a school population-based cross-sectional study

**DOI:** 10.3389/fendo.2023.1259172

**Published:** 2023-11-23

**Authors:** Jingyi Tang, Tingting Yu, Yanrui Jiang, Peng Xue, Huijun Kong, Cuilan Lin, Shijian Liu, Ying Tian

**Affiliations:** ^1^ Hainan Branch, Shanghai Children's Medical Center, School of Medicine, Shanghai Jiao Tong University, Sanya, China; ^2^ School of Public Health, School of Medicine, Shanghai Jiao Tong University, Shanghai, China; ^3^ Department of Developmental and Behavioral Pediatrics, Shanghai Children’s Medical Center, School of Medicine, Shanghai Jiao Tong University, Shanghai, China; ^4^ Department of Pediatrics, Qu Fu People’s Hospital, Qufu, Shandong, China; ^5^ BoAi Hospital of Zhongshan, Southern Medical University, Zhongshan, Guangdong, China; ^6^ Department of Environmental Health, School of Public Health, Shanghai Jiao Tong University School of Medicine, Shanghai, China

**Keywords:** sleep, early pubertal development, association, children, school

## Abstract

**Background:**

There is an increasing tendency toward early pubertal development, and sleep might be related to pubertal onset. We aimed to investigate the association of sleep duration and bedtime with early pubertal development.

**Methods:**

This was a cross-sectional study of 8,007 children (53.6% boys) from Qufu city, Shandong province and Zhongshan city, Guangdong province, China. Data on sleep duration and bedtime were obtained by questionnaire. Early pubertal development was the primary outcome and it was evaluated by the pediatrician according to Tanner staging. Logistic regression models were used to separately examine the association between sleep duration or bedtime and early pubertal development, controlling body mass index (BMI), dietary pattern, soft drink, feeding pattern and mother’s BMI.

**Results:**

In boys, short sleep duration was strongly related to early pubertal development [OR (95%CI): 4.26 (1.30, 13.94)], and this association was intensified after adjusted BMI, dietary pattern, soft drink, feeding pattern and mother’s BMI. In girls, OR (95%CI) was 1.62 (1.04, 2.51), and increased after controlling BMI. Bedtime was associated with early pubertal development on weekdays [OR (95%CI): 6.39 (1.54, 26.45) in boys and 1.93 (1.23, 3.05) in girls], but not on weekends [OR (95%CI): 2.49 (0.61, 10.21) in boys; 1.31 (0.76, 2.25) in girls].

**Conclusion:**

This study underscores the positive association between the risk of early pubertal development and insufficient sleep duration and late bedtime.

## Introduction

Early pubertal development is a common endocrine disease among children, characterized by early secondary sexual development and early menarche. The prevalence of early pubertal development is increasing worldwide, which is more frequent in girls. A South Korean study of registered insurance data discovered the prevalence of central precocious puberty in girls increased from 56.9/100,000 to 946.4/100,000, much higher than that in boys (increased from 2/100,000 to 30.1/100,000), from 2008 to 2014 (1). Similarly, the incidence of Danish girls climbed sixfold over 20 years (2). These alarming numbers call for attention to this global issue. Children with early pubertal development experience an earlier pubertal growth spurt, which exerts detrimental effects on the final height (3). Moreover, evidence suggests an association between early puberty and subsequent endocrine diseases (4).

Puberty is a complex stage that is easily affected by genetic and environmental factors. Mancini A pointed out that genes can explain 50-80% of abnormal timing of puberty onset (5). Besides, nutrients and diets in early life and childhood are linked to early puberty (6). Additionally, endocrine-disrupting chemicals (EDCs) pose risks to pubertal development. Studies corroborated that exposure to pesticides or phenols predisposes children to early pubertal development (7). Furthermore, the effects of living habits, such as insufficient sleep, have received keen attention.

Sleep is a multidimensional factor and necessitates multi-index evaluation, including sleep duration, sleep rhythm, and sleep regularity. Studies have demonstrated that sleep duration is related to obesity (8, 9). Given that obesity is a risk factor for precocious puberty, sleep might play a crucial role in development of early pubertal development. Several studies confirmed bedtime and sleep duration promote the timing of pubertal onset (10). However, evidence regarding this association is limited and there are racial and geographic differences in both pubertal timing and sleep. Therefore, we conducted a cross-sectional study to investigate the association of sleep and early pubertal development among Chinese children.

## Methods

### Study design and population

This school population-based cross-sectional study was conducted simultaneously in Zhongshan City, Guangdong Province and Qufu City, Shandong Province, China in 2019. According to a previous study performed in Guangdong Province, the prevalence of early pubertal development in children was 6.19%. A type I error (α) of 0.05, an expected prevalence (P) of 6.19%, a Z statistic for a level of confidence (1.96 for 95% confidence level) and a sampling error (δ) of 0.01*P indicated that at least 5,822 children should be recruited. We increased the sample size by 15% to deal with the possible loss of sample size due to unanticipated reasons such as non-response and incomplete data. Thus, a total of 6,850 children were required in this cross-sectional study.


$$N=Z_{\alpha /2}^{2}*P*\left(1-P\right)/\delta^{2}$$

A detailed study design was described in our previous article (11), we performed a stratified cluster random sampling method. In brief, we selected 21 schools from 7 of 9 urban districts and 5 of 26 suburban districts, and finally invited 13,170 children from all classes in grades 1-3. Among these, 9,788 (74.32%) agreed to participate and wrote the informed consent. This study was approved by the institutional review boards of Shanghai Children**’**s Medical Center, People**’**s Hospital of Qufu, and BoAi Hospital of Zhongshan.

According to the interest of outcome, we only included boys aged 6-9 and girls aged 6-8. We further excluded children lacking data on weight, height and puberty status, as well as children having a history of hormone use in the past six months. There were 8,007 participants left in the final analysis, including 4,295 boys and 3,712 girls.

### Demographic characteristics and sleep evaluation

We obtained the demographic characteristics using questionnaires filled by the guardians of students, including age, sex, feeding and dietary pattern, as well as information about parents and family income. The Pittsburgh Sleep Quality Index (PSQI), a self-rated questionnaire, was used to gather data on bedtime and sleep duration (12). In this study, late bedtime was deemed as after 22 PM. The National Sleep Foundation recommends 9-11 hours of sleep per night for children aged 6-13, sleep duration of fewer than 9 hours is deemed as insufficient sleep (13).

### Physical examination and evaluation of sexual characters

All participants were asked to keep barefoot and wear light clothes for weight and height measurement by pediatricians from local hospitals. Then BMI was calculated as weight (kg) divided by the square of height (m2). Based on criteria recommended by International Obesity Task Force (IOTF), children were classified as thinness, normal weight, overweight, and obesity according to the BMI cutoff 18.5, 25 and 30 (14). Then, girls were physically examined in a private room by qualified female pediatricians. Inspection and palpation were utilized to assess breast development. B-ultrasonic were combined to distinguish fat tissue and breast in obese girls. For boys, testicular volume was measured by a Prader testicular meter separately. The Tanner Staging was applied to evaluate children**’**s secondary sexual characteristics (15, 16). Early pubertal development is defined as girls whose breast development initiates before age 8; boys whose testicular enlarges (Testicular Volume ≥4mL) before age 9 (17).

### Statistical analysis

Continuous data, age and mother**’**s BMI, were all skewed distributions. Thus the Mann-Whitney U test was applied to compare the differences between early pubertal development and normal groups. The categorical data were presented with frequency, and the group comparisons were conducted using the Chi-square test. Logistic regression models were constructed to analyze the association between bedtime or sleep duration and early pubertal development. We confirmed the potential confounders based on previous studies. Models were initially adjusted for BMI category and subsequently for dietary pattern, soft drink, feeding pattern and mother**’**s BMI. The results were presented with odds ratio (OR) and 95% confidence interval (CI). All statistical analyses were conducted using SPSS 26.0 (IBM Corporation, Armonk, NY, USA). P-value**<**0.05 was deemed statistically significant.

## Results

### Participant’s characteristics

The characteristics of the included children were shown in **Table 1**, including 4,295 (53.6%) boys and 3,712 (46.4%) girls. The average age of boys and girls was 7.09 ± 0.79 and 7.13 ± 0.78, respectively. We identified 389 (4.9%) children who were diagnosed with early pubertal development. Of note, girls had a much higher prevalence of early pubertal development than boys (8.2% vs 2.0%, p<0.001). Evidently, boys diagnosed with early pubertal development were younger and suffered from higher BMI, shorter sleep duration, and later bedtime on both weekdays and weekends. Clearly, girls in two groups differed significantly in terms of age, BMI category, bedtime, dietary pattern, soft drink, feeding pattern, and mother’s BMI.

**Table 1:** Factors associated with early pubertal development (N= 8007).

Characters	Boys			Girls		
	Total N=4295	Early pubertal Development N (%)	*X^2^/Z* (*p*)	Total N=3712	Early pubertal DevelopmentN (%)	*X^2^/Z* (*p*)
Age	4295	86 (2.0)	-6.745 (**<0.001**)^*^	3712	303 (8.2)	-10.982 (**<0.001**)^*^
BMI			21.639 (**<0.001**)			153.916 (**<0.001**)
Thinness	591	6 (1.0)		676	28 (4.1)	
Normal weight	2627	46 (1.8)		2421	149 (6.2)	
Overweight	584	11 (1.9)		406	79 (19.5)	
Obesity	493	23 (4.7)		209	47 (22.5)	
Sleep duration			6.676 (**0.036**)			4.702 (0.095)
< 9 h	1323	33 (2.5)		1069	95 (8.9)	
9-10 h	2460	50 (2.0)		2165	181 (8.4)	
≥ 10 h	502	3 (0.6)		474	27 (5.7)	
Missing	10			4		
Bedtime						
Weekday			10.668 (**0.005**)			7.609 (**0.022**)
< 21 PM	455	2 (0.4)		431	23 (5.3)	
21-22 PM	2236	40 (1.8)		1918	146 (7.6)	
≥ 22 PM	1604	44 (2.7)		1363	134 (9.8)	
Weekend			10.635 (**0.005**)			12.283 (**0.002**)
< 21 PM	197	2 (1.0)		208	15 (7.2)	
21-22 PM	1203	12 (1.0)		989	56 (5.7)	
≥ 22 PM	2895	72 (2.5)		2515	232 (9.2)	
Dietary pattern			2.685 (0.261)			8.483 (**0.014**)
Meat mainly	1733	42 (2.4)		1287	128 (9.9)	
Vegetables mainly	485	9 (1.9)		470	32 (6.8)	
Balanced diet	2077	35 (1.7)		1955	143 (7.3)	
Soft drink			0.102 (0.749)			11.804 (**0.001**)
0	2569	50 (1.9)		2336	163 (6.6)	
≥1 times/week	1726	36 (2.1)		1376	140 (9.5)	
Feeding pattern			0.113 (0.945)			7.194 (**0.027**)
Breastfeeding	2299	45 (2.0)		2169	156 (7.2)	
Mixed feeding	598	13 (2.2)		483	50 (10.4)	
Formula feeding	1398	28 (2.0)		1060	97 (9.2)	
Mother’s menarche age			0.534 (0.465)			2.212 (0.137)
≥12 years	614	14 (2.3)		505	47 (9.3)	
< 12 years	2941	54 (1.8)		2615	193 (7.4)	
Missing	740			592		
Mother’s BMI	4295	86 (2.0)	-1.813 (0.070)^*^	3712	303 (8.2)	-2.905 (**0.004**)^*^

BMI, body mass index.

* The differences between two groups were assessed using the Mann-Whitney U test. The bold values highlight that the difference or association is statistically significant.

### Association of sleep and early pubertal development

As shown in **Table 2**, the Chi-square test confirmed the difference in sleep metrics between early pubertal development and normal puberty groups. Generally, boys showed distinct sleep duration and bedtime, whereas girls had different bedtime. In detail, the percentage of boys slept< 9 h in the early pubertal development group was slightly higher than that in the normal group (38.4% vs 30.7%, p=0.036). Furthermore, the percentage of boys slept ≥ 22 PM both on weekdays and weekends was higher, compared with that in the normal group (51.2% vs 37.1, p=0.005; 83.7% vs 67.1%, p=0.005). Similarly, girls diagnosed with early pubertal development had a propensity for later bedtime (44.2% vs 36.1%, p=0.005; 76.6% vs 67.0%, p=0.005).

**Table 2:** Characters of sleep in the children with early pubertal development and normal puberty (N= 8007).

Characters	Early pubertal development N=389 (%)	Normal puberty N=7618(%)	*X^2^* (*p*)
**Boys**	**86**	**4209**	
Sleep duration			6.676 (**0.036**)
< 9 h	33 (38.4)	1290 (30.7)	
9-10 h	50 (58.1)	2410 (57.4)	
≥ 10 h	3 (3.5)	499 (11.9)	
Missing		10	
Bedtime			
Weekday			10.668 (**0.005**)
< 21 PM	2 (2.3)	453 (10.8)	
21-22 PM	40 (46.5)	2196 (52.2)	
≥ 22 PM	44 (51.2)	1560 (37.1)	
Weekend			10.635 (**0.005**)
< 21 PM	2 (2.3)	195 (4.6)	
21-22 PM	12 (14.0)	1191 (28.3)	
≥ 22 PM	72 (83.7)	2823 (67.1)	
**Girls**	**303**	**3409**	
Sleep duration			4.702 (0.095)
< 9 h	95 (31.4)	974 (28.6)	
9-10 h	181 (59.7)	1984 (58.3)	
≥ 10 h	27 (8.9)	447 (13.1)	
Missing		4	
Bedtime			
Weekday			10.430 (**0.005**)
< 21 PM	23 (7.6)	408 (12.0)	
21-22 PM	146 (48.2)	1772 (52.0)	
≥ 22 PM	134 (44.2)	1229 (36.1)	
Weekend			10.645 (**0.005**)
< 21 PM	15 (5.0)	193 (5.7)	
21-22 PM	56 (18.5)	933 (27.4)	
≥ 22 PM	232 (76.6)	2283 (67.0)	

The bold values highlight that the difference or association is statistically significant.

### Association between sleep and early pubertal development by logistic regression

**Table 3** showed the results by regression models, using sleep duration or bedtime as explanatory variables singly. In boys, sleep duration was significantly associated with early pubertal development. Compared with boys who slept ≥ 10 h, the OR with 95%CI of PP for those who slept 9-10 h and< 9 h were 3.45 (1.07, 11.11) and 4.26 (1.30, 13.94), respectively. After adjusting for BMI, such association increased to 3.62 (1.12, 11.65) and 4.45 (1.36, 14.60), respectively. The results were still significant in the model controlling for all confounders [3.66 (1.14, 11.82); 4.44 (1.34, 14.65)]. In girls, the association between short sleep duration (<9 h) and early pubertal development was in the expected direction [OR (95%CI): 1.62 (1.04, 2.51)], and enhanced after controlling for BMI category [OR (95%CI): 1.79 (1.14, 2.80)]. In comparison, this association was slightly weak but more stable and credible among girls. We didn’t discover any association between bedtime on weekends and early pubertal development, while bedtime on weekdays might be a risk factor. Compared with boys who went to slept before 21 PM, the risk of early pubertal development increased 6.39-fold (95%CI: 1.54, 26.45) in groups who went to slept after 22 PM. This risk climbed to 7.22-fold after adjusting for BMI [OR (95%CI): 7.22 (1.74, 29.96)] and to 7.43-fold after adjusting for BMI, dietary pattern, soft drink, feeding pattern and mother’s BMI [OR (95%CI): 7.43 (1.78, 31.06)]. The association was attenuated in boys who went to slept during 21-22 PM [OR (95%CI): 4.40 (1.06, 18.28), adjusted for BMI; OR (95%CI): 4.49 (1.08, 18.69), adjusted for BMI, dietary pattern, soft drink, feeding pattern and mother’s BMI]. The risk of early pubertal development increased by 93% in girls who went to slept after 22 PM, compared to those who went to slept before 21 PM [OR (95%CI): 1.93 (1.23, 3.05)]. After adjusting for BMI or further adjusting for dietary pattern, soft drink, feeding pattern and mother’s BMI, this association was enhanced [OR (95%CI): 2.37 (1.48, 3.77); 2.06 (1.28, 3.31)]. In girls who went to slept during 21-22 PM, the risk of early pubertal development was increased to 1.60-fold [OR (95%CI): 1.60 (1.01, 2.54)] after controlling BMI.

**Table 3:** Associations between sleep and early pubertal development.

Variables	Boys (n=4295)			Girls (n=3712)		
	Unadjusted OR (95% CI)	Adjusted OR (95% CI)^a^	Adjusted OR (95% CI)^b^	Unadjusted OR (95% CI)	Adjusted OR (95% CI)^a^	Adjusted OR (95% CI)^b^
Sleep duration						
< 9 h	4.26 (1.30, 13.94)*	4.45 (1.36, 14.60)*	4.44 (1.34, 14.65)*	1.62 (1.04, 2.51)*	1.79 (1.14, 2.80)*	1.54 (0.97, 2.43)
9-10 h	3.45 (1.07, 11.11)*	3.62 (1.12, 11.65)*	3.66 (1.14, 11.82)*	1.51 (1.00, 2.29)	1.67 (1.09, 2.55)*	1.54 (1.00, 2.36)*
≥ 10 h	Reference	Reference	Reference	Reference	Reference	Reference
Bedtime						
Weekday						
< 21 PM	Reference	Reference	Reference	Reference	Reference	Reference
21-22 PM	4.13 (0.99, 17.13)	4.40 (1.06, 18.28)*	4.49 (1.08, 18.69)*	1.46 (0.93, 2.30)	1.60 (1.01, 2.54)*	1.52 (0.96, 2.42)
≥ 22 PM	6.39 (1.54, 26.45)*	7.22 (1.74, 29.96)**	7.43 (1.78, 31.06)**	1.93 (1.23, 3.05)**	2.37 (1.48, 3.77)***	2.06 (1.28, 3.31)*
Weekend						
< 21 PM	Reference	Reference	Reference	Reference	Reference	Reference
21-22 PM	0.98 (0.22, 4.42)	0.93 (0.21, 4.19)	0.94 (0.21, 4.26)	0.77 (0.43, 1.39)	0.83 (0.45, 1.51)	0.85 (0.47, 1.56)
≥ 22 PM	2.49 (0.61, 10.21)	2.61 (0.63, 10.72)	2.66 (0.65, 11.00)	1.31 (0.76, 2.25)	1.56 (0.90, 2.71)	1.45 (0.83, 2.53)

OR, Odds Ratio; CI, confidence interval.

*,<0.05; **,<0.01; ***,<0.001.

^a^ Adjusted for BMI; ^b^ Adjusted for BMI, Dietary pattern, Soft drink, Feeding pattern and Mother’s BMI. The bold values highlight that the difference or association is statistically significant.

## Discussion

In this study, we investigated the association between early pubertal development and sleep, including sleep duration and bedtime. We provided evidence that sleep duration had a negative relationship with early pubertal development in boys and girls. Besides, the risk of early pubertal development climbed profoundly among children who had later bedtime. Our findings suggested that insufficient sleep and late bedtime posed a risk for the development of early puberty.

### Late bedtime and insufficient sleep time among children

Sleep is the cornerstone of a healthy body and mind. Unfortunately, late bedtime and insufficient sleep have become ubiquitous among children. In the present study, girls and boys aged 6-9 had a definite trend of late bedtime and short sleep duration. Approximately 30% boys and girls slept for less than 9 h. In addition, 37.3% boys and 36.7% girls slept after 22 PM on weekdays. On weekends, this behavior is particularly pronounced. Late bedtime was discovered in 67.4% boys and 67.8% girls, respectively. This aligns with a series of previous studies, although there were substantial discrepancies in age and countries. An investigation in China found that the medium bedtime of children aged 9.25 years during weekdays and weekends were 22:22 PM and 22:56 PM, respectively (18). An American study reported children aged 4-13 years exhibited a delay in bedtime routine (>22 PM) (19). In parallel with the late bedtime, a secular trend of decline in sleep duration in children is evident. In a cross-sectional study with large samples, the mean (SD) sleep duration of children was 9 h (1.11) (20). A pilot study in Spain found that only 9.4% school-aged children slept for more than 9 h on weekends, whereas only 5.4% on weekdays (21). Preliminary evidence endorsed that short sleep duration is partly due to the late bedtime, particularly on weekdays (19). Indeed, delayed bedtime, coupled with an early school start time, contribute to insufficient sleep duration among school children. However, late bedtime disturbs circadian rhythm, mainly affecting sleep architecture rather than duration (22). Several studies supported that both short sleep duration and late bedtime are linked to the timing of puberty onset (10, 23).

### Mechanisms linking sleep to early pubertal development

There is no consensus on the mechanism linking sleep with early pubertal development, some possible explanations have been proposed. Insufficient sleep duration and late bedtime are risk factors for obesity, and obesity increases risk of earlier puberty, so obesity might play a mediated role. Previous studies suggested less sleep duration boosts food intake mainly by affecting appetite-regulating hormones like leptin and ghrelin. Among children and adults, the relationship between sleep and leptin levels was inconsistent. Children who sleep less tend to have higher leptin levels (24, 25). Leptin, a hormone that suppresses appetite, cannot explain the fact that children with less sleep are prone to be obese. Building on these findings, another putative mechanism gains more support. Bedtime and sleep duration can alter brain functions involved in food consumption, relating to emotion rather than satiety (26). The food reward system, consisting of food pleasure and food desire, encourages overeating and changes children’s food preferences. An experimental study among adolescents corroborated that sleep restriction led to a preference for energy-dense/nutrient-poor diets, as well as a readiness to pay more for them (27). Similarly, by interfering in judging the value of food, this reward system can be activated by late bedtime (22). Food intake driven by the reward rather than metabolic need multiplies the risk of obesity (28), predisposing children to early pubertal development (29). In addition, insufficient and late sleep increase time and opportunity to eat, especially high-sugar and ultra-processed foods (26, 30). Late bedtime is frequently accompanied by irregular nighttime eating (31). A review pointed out that the alteration in sleep rhythm may change the metabolic efficacy, which might explain why late bedtime predisposes children to be obese (22).

Preliminary evidence revealed that sleep can influence Hypothalamus-Pituitary-Gonad (HPG) axis. As previously stated, short sleep duration induces higher leptin in children. Leptin can stimulate the release of kisspeptin (32). Kisspeptin acts on its receptor on gonadotropin-releasing hormone (GnRH) neurons to promote the secretion of GnRH and in turn activate the pubertal development progress (33). Melatonin, regulated by dark-light, might be a vital pathway linking sleep rhythm and the timing of puberty. Exposure to electronic screens is evidenced to delay the circadian phase by up to 1.5 h (34). Late bedtime exposes children to room light immoderately, which disrupts melatonin signaling and suppresses melatonin secretion (35). In mice, melatonin can hinder testis development by directly acting on its receptors in testis, as well as by suppressing GnRH release (36). Despite some debates (37, 38), several studies endorsed the connection between melatonin and human puberty. A human investigation observed a sudden drop in melatonin concentrations as development progressed, indicating its vital role in the initiation of puberty (39). Taken together, further prospective studies are needed to confirm the speculation that low melatonin levels evoked by late bedtime can cause early pubertal development.

### Strengths and limitations

We noted that this is the first study based on large-scale school populations to investigate the association between sleep and early pubertal development. The participants were from two cities in southern and northern China, narrowing the geographical impact on the findings. Also, the participants were sampled using a multistage stratified cluster random sampling approach to guarantee representation. Besides, the sleep questionnaires were filled in under guidance at school, giving participants the chance to ask for explanations, and thus improving the completeness and preciseness. Moreover, to ensure the accuracy of puberty status assessment, secondary sexual characteristics were assessed by professional pediatricians. BMI, feeding and dietary pattern are all risk factors for early pubertal development, according to prior research. We built different regression models adjusted for these potential confounders to ensure the robustness of the conclusion.

However, some limitations merit attention. First, the cross-sectional study design hindered our ability to determine the causal relationship between sleep and early pubertal development. Large cohort studies are required to support our findings. Second, data on bedtime and sleep duration from questionnaires inevitably caused recall bias. But it was a practical and cost-effective approach in studies with large samples. Third, the puberty status was a one-time screening. Thus, girls with transient breast development cannot be ruled out. To address this issue, we have conducted a follow-up study. Among the 303 girls with early pubertal development in this study, only 32 (10.56%) were subsequently identified as transient breast development, resulting in a remaining prevalence of early pubertal development of 7.3%. However, the puberty status was only evaluated using the Tanner stage, thus, we cannot make a definitive diagnosis of precocious puberty. We have tested basal hormones including LH/FSH/E2 among 887 girls, and the cutoff 0.3 IU/L of basal LH was used. Only about 10% of these girls were diagnosed with true precocious puberty by basal determination of LH > 0.3 IU/L. In the future survey, we will inform the children who are screened as potential cases with early pubertal development to accept a free clinical examination in our cooperating hospital. We believe it is a relatively cost-effective method to improve the accuracy of the assessment of pubertal status. Fourth, the children who were willing to participate in this study might pay more attention to their lifestyle and have healthier sleep habits. As a consequence, the connection between sleep and early pubertal development might be attenuated. Fifth, the marginal associations from logistic regression models called for a cautious interpretation of the relationship between sleep duration and early pubertal development. In comparison, the association was stable for bedtime and early pubertal development, especially in girls. Lastly, some confounders in the adjusted models might not be reliable, for instance, data on feeding patterns and dietary patterns were all self-reported. Nevertheless, we were able to adjust for the reliable BMI, as height and weight were clinically measured.

## Conclusion

In conclusion, shorter sleep duration and late bedtime appear to be modifiable determinants of early pubertal development. Further cohort studies are needed to verify the causal relationship between sleep and early pubertal development, as well as to delve deeper into the underlying mechanisms.

## Data availability statement

The raw data supporting the conclusions of this article will be made available by the authors, without undue reservation.

## Ethics statement

The studies involving humans were approved by Institutional review boards of Shanghai Children’s Medical Center, People’s Hospital of Qufu, and Bo Ai Hospital of Zhongshan. The studies were conducted in accordance with the local legislation and institutional requirements. Written informed consent for participation in this study was provided by the participants’ legal guardians/next of kin.

## Author contributions

JT: Formal Analysis, Investigation, Methodology, Writing – original draft, Data curation. TY: Data curation, Formal Analysis, Investigation, Methodology, Writing – original draft. YJ: Data curation, Formal Analysis, Investigation, Writing – original draft. PX: Data curation, Investigation, Writing – original draft. HK: Investigation, Writing – original draft. CL: Investigation, Writing – original draft. SL: Conceptualization, Funding acquisition, Writing – review & editing. YT: Conceptualization, Writing – review & editing.
